# The UK Crop Plant Bioinformatics Network (UK CropNet)

**DOI:** 10.1002/1097-0061(200012)17:4<335::AID-YEA49>3.0.CO;2-7

**Published:** 2000

**Authors:** Keith Bradnam, Sean May

**Affiliations:** Nottingham Arabidopsis Stock Centre (NASC) School of Biosciences, Plant Science Division University of Nottingham Life and Environmental Sciences Building, University Park Nottingham NG7 2RD UK

## Abstract

UK CropNet currently provides a range of databases (and database-mining tools) to the plant community that are all freely accessible through our website (http://ukcrop.net/). Recent upgrades have meant that we can now expand the range of available facilities (e.g. addition of new databases) whilst also strengthening and improving access to existing services (e.g. providing a BLAST search facility against sequences in our databases). This article will briefly outline these and other new developments in our service.


**Yeast**
*Yeast* 2000; **17**: 335–338.

**Website Update**

### The UK Crop Plant Bioinformatics Network (UK CropNet)

http://ukcrop.net

Keith Bradnam and Sean May
*Nottingham Arabidopsis Stock Centre (NASC), School of Biosciences, Plant Science Division, University of Nottingham, Life & Environmental Sciences Building, Nottingham, University Park, NG7 2RD, UK*

**Correspondence to:*
*K. Bradnam, Nottingham Arabidopsis Stock Centre (NASC), School of Biosciences, Plant Science Division, University of Nottingham, Life and Environmental Sciences Building, University Park, Nottingham NG7 2RD, UK*

Received: 25 September 2000
Accepted: 26 September 2000

### Abstract

**UK CropNet currently provides a range of databases (and database-mining tools) to the plant community that are all freely accessible through our website (**http://ukcrop.net/**). Recent upgrades have meant that we can now expand the range of available facilities (e.g. addition of new databases) whilst also strengthening and improving access to existing services (e.g. providing a BLAST search facility against sequences in our databases). This article will briefly outline these and other new developments in our service. Copyright © 2000 John Wiley & Sons, Ltd.**

### Faster machine, faster access

Several practical improvements are immediately available due to the increased memory, processing power, and disk space on our new UK CropNet server. For example, we can now handle multiple database connections more efficiently, providing an increase in the apparent 'speed' to the end user. This is most likely to be noticed when many users are connecting simultaneously.

Our increased disk space now allows UK CropNet to mirror many more databases from other sources (principally from Cornell University). The eighteen existing ACEDB plant-related databases (five from CropNet, thirteen mirrored from Cornell) are joined by an additional thirteen databases. These include many new databases for model organisms (e.g. MaizeDB) and other globally important plant species (e.g. CassavaDB). We now also have several databases that are quite atypical of most ACEDB databases, i.e. they don't contain any sequences or maps. Such a database is EcoSys, a database that contains details on the natural environmental conditions (temperature, soil pH, precipitation, etc.) for nearly 900 different taxa, arranged by family and genus.

Database searching is now much faster which has made it possible to develop a prototype multiple-database-search facility (see: **http://ukcrop.net/perl/ace/mgrep/** or **http://ukcrop.net/db.html**). This facility will allow a user to perform a text-based query search against any (or all) of our databases (Figure 1), i.e. a user can search for the name of a gene of interest across all of the databases at our site.

### Improvement to web-based database interfaces

Accessing an ACEDB-style database via the web can sometimes prove unsatisfactory when compared to using the database on a local workstation. This is because the functionality of a complex database program such as ACEDB is compromised by the restrictions of what you can and cannot do within the confines of an HTML web page. To address this, we have introduced a number of new modifications and tweaks to the standard package of

Copyright © 2000 John Wiley & Sons, Ltd.

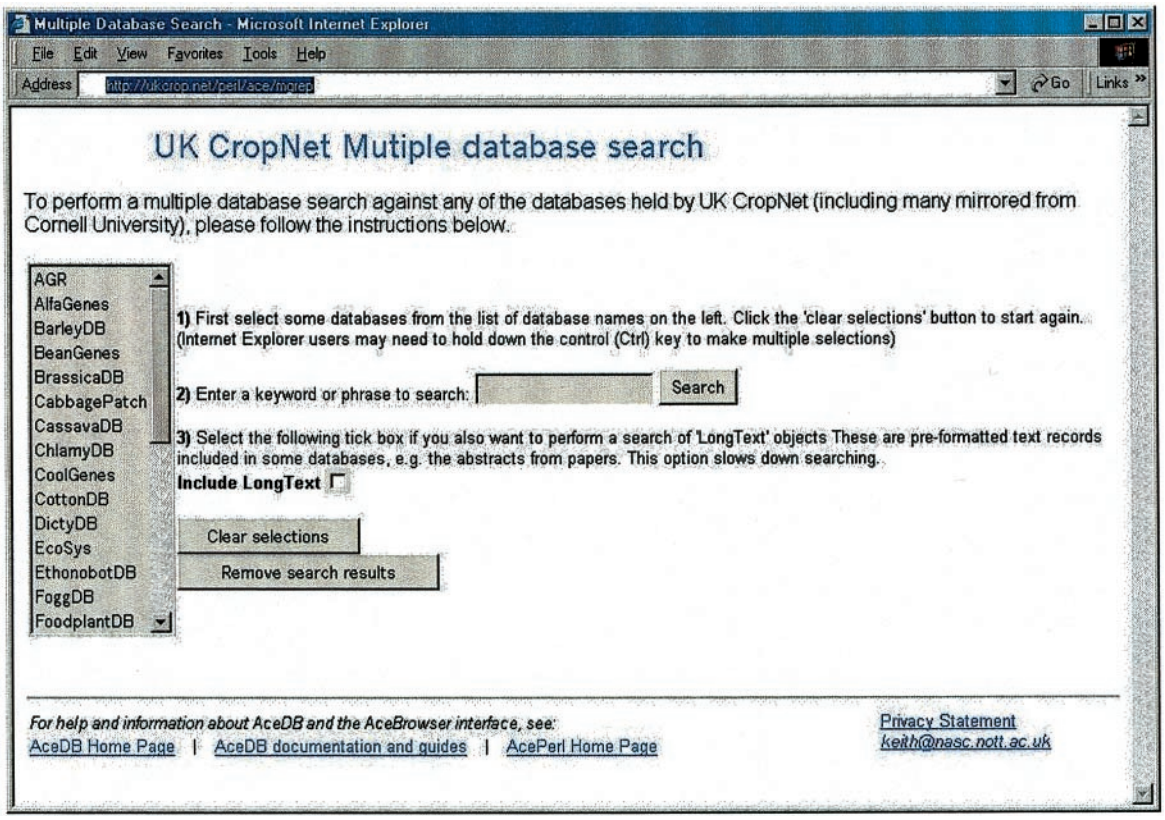

**Figure 1.** The multiple database search tool

ACEDB web interfaces (collectively known as 'AceBrowser'). At a very simple level, some of these 'tweaks' involve nothing more than the simple rewording of much of the default AceBrowser text to something that is (hopefully) more lucid and intuitive to the end user (particularly when English might not be their first language). At a more advanced level, we have refined many of the AceBrowser interfaces and, where necessary, developed and added completely new interfaces.

For instance, several of the CropNet databases (AGR, BrassicaDB, MilletGenes) are now linked to a Java based map display (Pairwise Comparative Map, PCM) that offers vastly improved functionality over the standard ACEDB web interface for viewing maps. Users can now quickly resize, zoom, and invert maps and compare multiple maps in the same, interactive, display (where multiple maps exist).

Other modifications to the ACEDB web interfaces include increasing the amount of information available within graphic displays of sequences (useful when viewing sequences which have a lot of associated homology information from 'BLAST hits') and providing access to the ACEDB 'Table-Maker' facility for data organisation through the standard web interface. It has also been possible to configure the database displays to allow for inter-database linking, e.g. a BLASTX homology from a nucleotide sequence in BrassicaDB can now be linked to the corresponding protein sequence in AGR.

One of the major modifications to the AceBrowser interface has been the addition of a BLAST search facility. When browsing any (sequence-containing) database, users can now access a BLAST page (Figure 2), which provides a search facility for any of these databases. BLAST databases are updated each night to reflect any new sequence additions. For Arabidopsis, it has also been possible to create multiple BLAST databases to reflect the fact that the Arabidopsis genome sequence is nearly complete. Users can thus search against all Arabidopsis DNA ($\sim$250,000 sequences)

Copyright © 2000 John Wiley & Sons, Ltd.

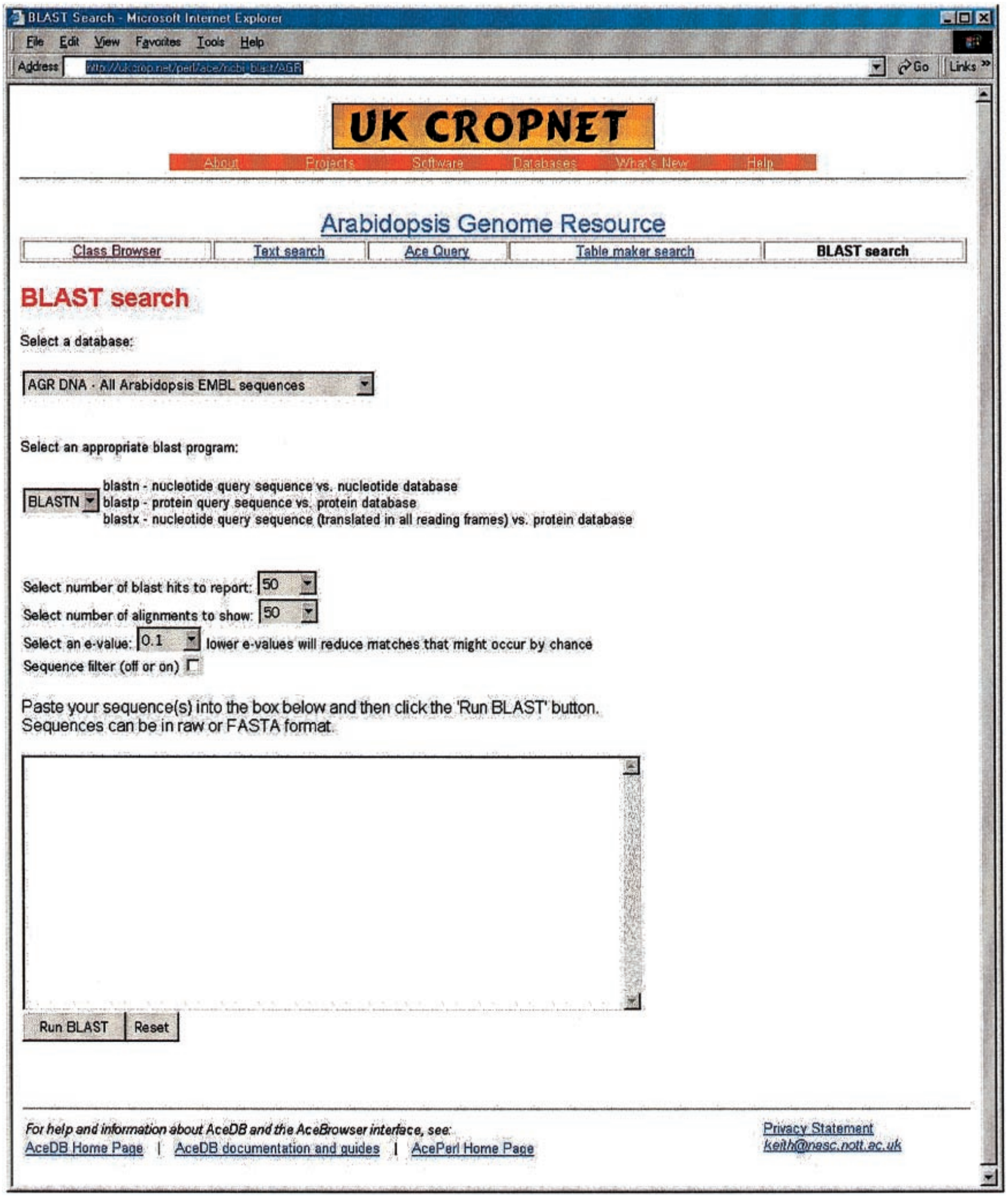

**Figure 2.** The BLAST search page for AGR

or only against those sequences that comprise the Arabidopsis Genome Initiative (AGI) genome sequence. Even more specifically, users can search against AGI sequences that have been mapped to each of the five chromosomes. Currently, a choice of BLASTN, BLASTP, and BLASTX is available depending on whether protein or nucleotide sequences exist for a given database.

Early in 2001 we will be introducing a generic Java based access tool developed within UK CropNet as an alternative method for browsing our databases. This tool is 'GFace' and provides an

Copyright © 2000 John Wiley & Sons, Ltd.

opportunity to utilise realtime manipulations of data via the web that eliminates many of the refresh issues and some of the lack of functionality that has been inevitable through simple HTML based browser access. GFace has been specifically designed to incorporate all of UK CropNet's Java tools (e.g. PCM mentioned above) and is also designed to integrate with tools (under development) for the cross-interrogation of ACEDB style databases with other plant database formats such as Oracle.

On a final note, we are constantly striving to make our service as accessible and powerful for the widest possible scientific audience. This ranges from making our databases easier to use and the data more conveniently visualised, to incorporating 'on-the-fly' translations for several European languages via 'babel-fish' from alta-vista. As part of this endeavour, we welcome any and all comments and criticisms (preferably in English) that may help to improve and develop UK CropNet in the future.

### Acknowledgements

All screen views are reproduced with the kind permission of Dr Sean May, Director, Nottingham *Arabidopsis* Stock Centre (NASC).

---

*Comparative and Functional Genomics* publishes Website Reviews. To keep you up-to-date with changes to these sites of interest, we also bring you Website Updates. These Website Updates are invited, and represent an explanation and personal critical appraisal of new tools and services which are to be, or have recently been, added to a site that we have previously reviewed..

The Genomics Website at Wiley www.wiley.co.uk/genomics

The **Genomics** website at Wiley is a new and **DYNAMIC** resource for the genomics community, offering **FREE** special feature articles and new information **EACH MONTH**.

Find out more about *Comparative and Functional Genomics*, and how to view all articles published this year **FREE OF CHARGE**!

Visit the **Library** for hot books in Genomics, Bioinformatics, Molecular Genetics and more.

Click on **Primary Research** for information on all our up-to-the minute journals, including: *Genesis*, *Bioessays, Gene Function and Disease*, and the *Journal of Gene Medicine*.

Let the **Genomics** website at Wiley be your **guide** to genomics-related web sites, manufacturers and suppliers, and a calendar of conferences.

WILEY

1516

Copyright © 2000 John Wiley & Sons, Ltd. 

